# Multiscenario Land Use Change Simulation and Its Impact on Ecosystem Service Function in Henan Province Based on FLUS‐InVEST Model

**DOI:** 10.1002/ece3.71111

**Published:** 2025-03-13

**Authors:** Jincai Zhang, Ling Li, Qingsong Li, Weiqiang Chen, Junchang Huang, Yulong Guo, Guangxing Ji

**Affiliations:** ^1^ College of Resources and Environmental Sciences Henan Agricultural University Zhengzhou Henan China

**Keywords:** comprehensive CES index services ecosystem, ecosystem services, FLUS model, Henan Province, InVEST model, land use change

## Abstract

Studying the impact of future land use changes on regional ecosystem services (ES) is crucial for sustainable development planning in the region. However, there is a lack of research specifically targeting Henan Province under different future scenarios. Therefore, this study simulates four ES functions—water yield (WY), carbon storage (CS), habitat quality (HQ), and nutrient delivery ratio (NDR)—for the historical period in Henan Province. It also constructs a Comprehensive Ecosystem Service (CES) Index. Additionally, the study predicts the spatial and temporal distribution characteristics of various ES and CES under two different Shared Socioeconomic Pathways (SSP) scenarios for the future. The results of the study showed that: (1) The high simulation accuracy of the FLUS model indicates that the FLUS model is suitable for land use simulation in the study area. (2) Under the SSP2‐4.5 scenario, the area of construction land expansion is the largest, and the HQ, CS, water production capacity, and water purification capacity of Henan Province all decrease. Under the SSP5‐8.5 scenario, the area of cultivated land increased the most, and all three decreased except for the water production capacity, which increased. (3) Under the SSP2‐4.5 scenario, the area of CES decline is the largest, and the severe decline in CES mainly occurs in areas where forest land is encroached upon by urban land, followed by areas encroached upon by rural settlements, and the encroachment of arable land by construction land leads to a mild decline in CES, which accounts for the largest proportion of the area. Under the SSP5‐8.5 scenario, Henan Province has the largest area of CES rise, and most of it is dominated by mild rise, but the mean CES in 2050 is still lower compared to 2020. The results of the study can provide a reference basis for the formulation of sustainable development policies in Henan Province and provide new ideas for the study of the impacts of land use change on ES under different scenarios in the future.

## Graphical abstract

The InVEST model was used to calculate the ecosystem service functions of the four modules of water yield, carbon storage, HQ, and nutrient delivery ratio, and to construct a comprehensive index of ES (CES) in Henan Province from 2000 to 2050. Finally, the temporal and spatial changes of various ecosystem service functions and CES were analyzed.

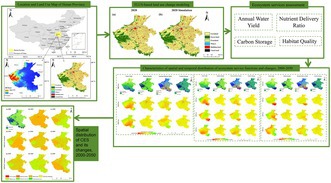

## 1 Introduction

Ecosystem services (ESs) refer to the benefits that humans derive directly or indirectly from ecosystems, and mainly include four types of services: provisioning, regulating, cultural and supporting services (Costanza et al. 1998; Ouyang et al. 1999). Natural capital, mainly in the form of ESs, facilitates human social development and enhances human well‐being, providing a resource and environmental foundation for human survival and development (Kremen and Ostfeld 2005; Summers et al. 2012; Bai 2011). However, the increase in population and unlimited human production and business activities have caused serious damage to the ecological environment, resulting in a series of major natural disasters such as the increased frequency and intensity of extreme weather and fires (IPCC 2014; Mahecha et al. 2017; Ummenhofer and Meehl 2017), which has led to a global scale of ecological system structure and functioning at the global scale. According to the United Nations “Ecosystem Services and Human Well‐being” (MA) program, more than 60% of the world's ecosystem services have declined to varying degrees, and this downward trend is likely to continue, which is a serious obstacle to sustainable human development (Briones‐Hidrovo et al. 2020; Mi et al. 2021). Statistics show that ecosystem function can create up to 15 trillion pounds of value for human beings (Wu et al. 2020), however, under the irrational use of human beings, nearly two‐thirds of this value has been lost, and problems such as soil erosion, land sanding, and a significant decline in biodiversity have led to a decline in ecological function and undermined the sustainable development of the ecological environment at the same time (Jiang et al. 2021; Chatanga et al. 2020), it is urgent to assess and improve ESs (Yang et al. 2020; Fu et al. 2020).

Early ecosystem service assessment suffered from the problems of a single research object and one‐sided research conclusions (Wang et al. 2013; Deng et al. 2019). In order to be able to better quantify ESs, some assessment models based on complex theories and research results have emerged, such as InVEST (Benra et al. 2021), the ARIES (Villa et al. 2009) and SolVES (Kim et al. 2017; Zhao et al. 2019) among other models. Compared with other models, the InVEST model is more maturely developed, has the advantages of easy access to driving data, easy parameter adjustment, visualization of results, and is in the stage of continuous updating and improvement, so it has been widely used (Li et al. 2023, 2024; Yue et al. 2023) and has achieved a better response all over the world. For example, Zhang et al. (2022) used InVEST's carbon stock, HQ, water yield (WY), and sediment transport ratio models to assess the conservation effects inside and outside the Qinghai‐Tibet Plateau National Nature Reserve. The InVEST model has been widely used at different research scales, and its reliability has been verified, e.g., for the urban agglomeration of the Jing‐Guan‐Zhong Plain, China (Yang et al. 2022), on the spatiotemporal dynamics of the Loess Plateau in China (Chen et al. 2022) and on the dynamics of soil and water conservation and water quality purification in the Koshi River Basin in Nepal (Yigez et al. 2023). Several practical cases have demonstrated that the InVEST model can better reflect the ecological processes in different ecosystems and well assess the spatial characteristics of ESs.

Land use/cover change (LUCC) is the most important factor affecting ESs (Rao et al. 2018; Wang et al. 2018), and certain ecosystem service functions are closely related to land use type changes. In recent years, many scholars have explored the impacts of land use change on ecosystem functions such as soil and water conservation, biodiversity, and HQ from different research perspectives. For example, Liu et al. (2015) showed that the increase of grassland, watershed, and forest land has a positive effect on the water environment, while the increase of agricultural and construction land has an almost equal negative effect on the water environment. Li et al. (2013) showed that the reduction of farmland and the expansion of forests improved the soil conservation service, while the increase of forests improved the carbon sequestration service but weakened the water resource supply service, and the expansion of construction land improved the soil conservation service, and the increase of forests improved the water resource supply service. The expansion of building land will greatly reduce the function of water purification. The research mentioned above only covers the impact of land use change on ecosystems during historical periods. To better study the future impact of land use change on ecosystems, Thomas et al. (2024) proposed the use of endogenous value coefficients in the assessment of future ecosystem service values. Additionally, scholars have utilized models such as CA‐Markov (Liu et al. 2021), CLUE‐S (Li et al. 2021), and the FLUS model (Wang et al. 2020) to simulate future land use conditions, thereby modeling future ecosystem functions. Chen et al. (2024) have employed the CA‐Markov and InVEST models to simulate the spatial flow of future ESs. Their study indicates that vegetation coverage is the primary driving force for soil conservation. Compared to the CA‐Markov model, the FLUS model (Liu et al. 2017) is better suited for simulating complex land use changes in multiple scenarios, across various scales, and with high precision (Liang et al. 2018), and it has been more widely applied. For instance, Ma et al. (2024) combined the FLUS and InVEST models to evaluate the relative contributions and comprehensive impacts of water‐related ecosystem functions in Southwest China, finding that land use and climate change have a comprehensive impact on future ESs. Li et al. (2020) also used the FLUS and InVEST models to investigate the effects of land use change on carbon storage (CS), flood regulation (FR), and soil conservation (SC) in the Beijing Ecological Protection Area of China. These studies suggest that the combination of FLUS and InVEST models is more suitable for simulating future land use and ecosystem service functions in multiple scenarios.

Henan Province is China's most populous and agricultural province, with 6% of the country's arable land and 10% of its population (Zhang 2021). It is the main producing area for wheat, corn, and other grain crops in China (Wang et al. 2023) and is a key region for realizing China's rural revitalization strategy. However, most areas in Henan Province are still ecologically fragile (Song and Yang 2022), with issues such as forest structure problems, land desertification, soil erosion, and agricultural non‐point source pollution (Huang et al. 2021). Previous studies have focused on the single functions of ecosystems in Henan Province (Ren et al. 2022; Fan et al. 2023) or on CES valuation of an area in Henan Province (Niu et al. 2022; Ji et al. 2021). No scholars have yet used InVEST to integrate multiple indicators to comprehensively assess the impacts of ecological functions and their spatial and temporal changes in Henan Province. Henan Province has reached a forest coverage rate of 25.47%, but this coverage is not uniformly distributed across the region. Similarly, while the total water resources in the province are adequate, there is a disparity in their spatial distribution. The amount of carbon stored is linked to land use patterns, and there is a spatial mismatch between the supply and demand of carbon sequestration services (Li et al. 2022). In Henan Province, the issue of non‐point source pollution is severe (Gao et al. 2024). Evaluating the nitrogen load is essential for the government to develop effective strategies for enhancing water quality. Considering the current situation in Henan, the assessment of HQ, WY, carbon storage (CS), and nitrogen export is vital for the protection of biodiversity, management of water resources, reduction of carbon emissions, and improvement of water quality.

Therefore, this paper uses the FLUS model to check the accuracy of land use data in Henan Province and then simulates the multi‐scenario land use data from 2030 to 2050 using the FLUS model. After that, the InVEST model was used to quantify four ESs: CS, WY, HQ, and nutrient delivery ratio (NDR) for the period 2000–2050, and a hierarchical analysis was used to construct a comprehensive ecosystem services index (CES) for comparing the overall levels of multiple ESs. Finally, the impacts of land use drivers on CES in Henan Province are explored using geoprobes, and changes in multiple ESs and their total supply in Henan Province are further analyzed for multiple scenarios for 2000–2020 and 2030–2050. It is hoped that this will provide information for future land use decisions in Henan Province, a theoretical basis for ecological revitalization in Henan Province, and a reference for ensuring ecological security in Henan Province.

## 2 Study Area and Data Sources

### 2.1 Overview of the Study Area

**FIGURE 1:**
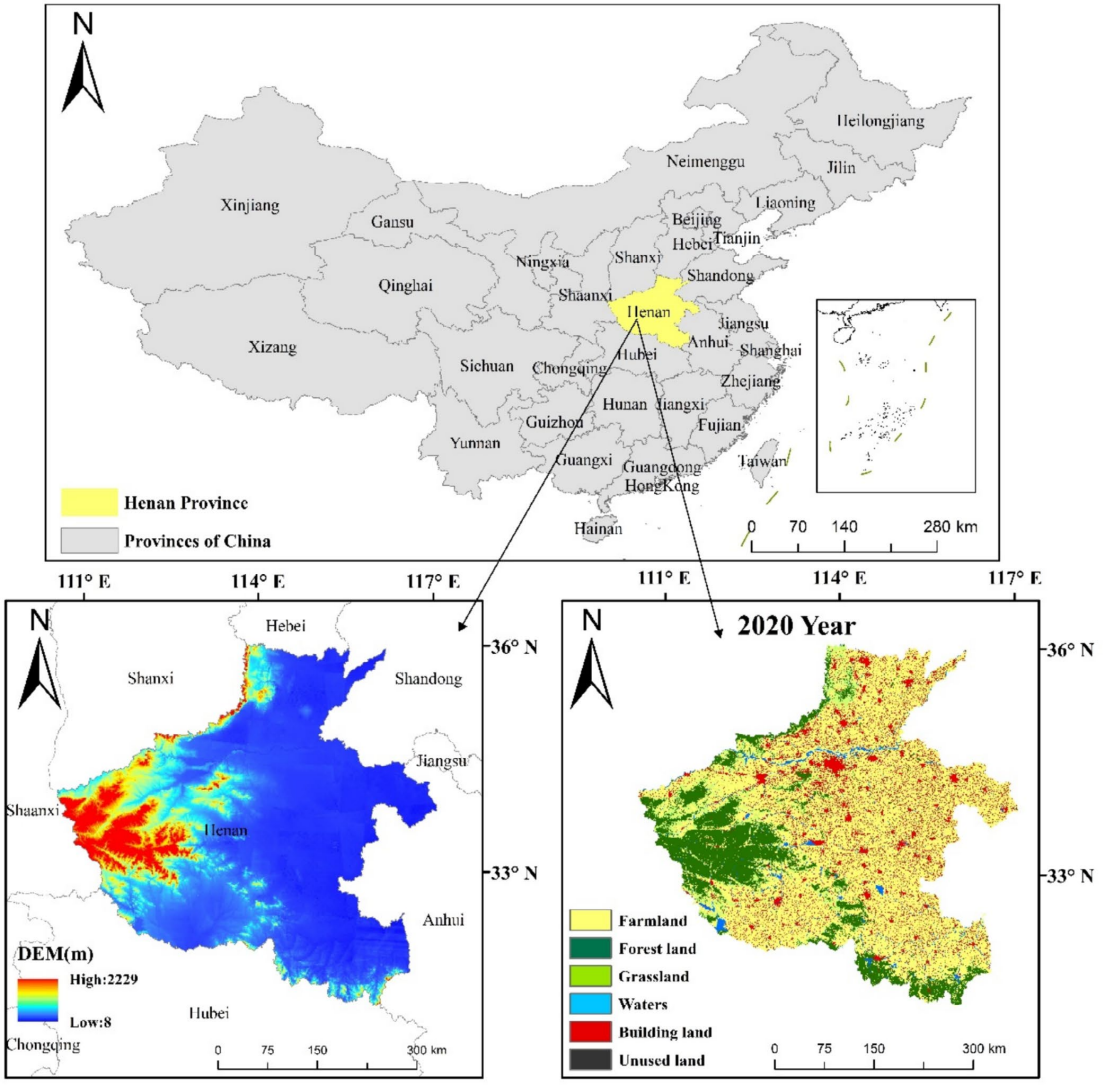
Location and land use map of Henan Province.

The geographical location of Henan Province is shown in Figure 1. Henan Province is located in the middle and lower reaches of the Yellow River in east‐central China, south of the North China Plain. It is located at longitude 110°21′–116°39′ E and latitude 31°23′–36°22′ N, with a straight‐line distance of about 580 km from east to west and 550 km from north to south, and the total area of the province is 167,000 km^2^. The topography of Henan Province presents the trend of looking north to south and bearing east to west; the terrain is high in the west and low in the east. The north, west, and south are bounded by the Taihang Mountains, Mount Fuyao, Mount Tongbai, and Mount Dabie along the provincial boundary in a semiannular distribution, in the middle and east of the Yellow River and Huaihai alluvial plain, and the southwestern part of the Nanyang Basin. The plains and basins, mountains, and hills account for 55.7%, 26.6%, and 17.7% of the total area, respectively. Most of Henan Province is located in the warm temperate zone; the south crosses into the subtropics, belonging to the continental monsoon climate of the transition from the northern subtropics to the warm temperate zone, but it also has the characteristics of the transition of the climate from east to west from the plains to the hilly and mountainous areas, with four seasons, rain and heat at the same time, complex and varied meteorological disasters that occur frequently. The average annual temperature of the province from south to north ranges from 10.5°C to 16.7°C, and the average annual precipitation ranges from 407.7 to 1295.8 mm.

### 2.2 Data Source and Description

The data used in this study and its sources are shown in Table 1. In this study, the land use data for the historical periods (2000, 2010, and 2020) were further categorized into six types based on the original data: agricultural land, forest land, grassland, wetland, building land, and unused land. The global 0.25° × 0.25° Land Use Harmonization 2 (LUH2) dataset was downscaled to obtain predicted quantities of various land use types for the future periods of 2030, 2040, and 2050 under two SSP scenarios (SSP2‐4.5 and SSP5‐8.5). The SSP scenarios SSP2‐4.5 and SSP5‐8.5 are briefly described as follows: In the SSP2‐4.5 scenario, socioeconomic factors follow their historical trends without significant changes, and CO_2_ emissions start to decline in the mid‐21st century. Under this scenario, the temperature is expected to rise by 2.7°C by the end of the century. SSP5‐8.5 is a more aggressive scenario where CO_2_ emission levels double by 2050, with rapid global economic growth. By 2100, the global average temperature is projected to be 4.4°C higher in this scenario.

**TABLE 1:** Data name and source.

Data name	Spatial resolution	Time	Source
Land use data	30 × 30 m	2000, 2010, and 2020 year	https://www.resdc.cn/
Digital Elevation Model	1000 × 1000 m	2020 year	https://www.gscloud.cn/
Oxygen content of soil root	1000 × 1000 m	2020 year	http://webarchive.iiasa.ac.at/Research/LUC/External‐World‐soil‐database/
Optimum soil tilth	1000 × 1000 m	2020 year	http://webarchive.iiasa.ac.at/Research/LUC/External‐World‐soil‐database/
Soil salt salinity	1000 × 1000 m	2020 year	http://webarchive.iiasa.ac.at/Research/LUC/External‐World‐soil‐database/
Plant Available Water Content	1000 × 1000 m	2020 year	https://data.isric.org/geonetwork/srv/eng/catalog.search#/metadata/e33e75c0‐d9ab‐46b5‐a915‐cb344345099c
Precipitation	1000 × 1000 m	2000, 2010 and 2020 year	https://www.resdc.cn/
Evapotranspiration	1000 × 1000 m	2000, 2010, and 2020 year	http://www.geodata.cn/
Root Restricting Layer Depth	1000 × 1000 m	2020 year	https://doi.org/10.1038/s41597‐019‐0345‐6
SSP scenario Land use data	0.25° × 0.25°	2030, 2040, and 2050 year	https://luh.umd.edu/data.shtml/
SSP scenario rainfall data	1000 × 1000 m	2030, 2040, and 2050 year	https://esgf‐node.llnl.gov/projects/cmip6/
SSP scenario evaporation data	1000 × 1000 m	2030, 2040 and 2050 year	https://esgf‐node.llnl.gov/projects/cmip6/
Soil type	1000 × 1000 m	2020 year	https://www.resdc.cn/Default.aspx
Normalized Difference Vegetation Index	30 × 30 m	2020 year	https://www.resdc.cn/Default.aspx
Night light data	1000 × 1000 m	2020 year	https://www.resdc.cn/Default.aspx
Population density	1000 × 1000 m	2020 year	https://landscan.ornl.gov/
Temperature	1000 × 1000 m	2020 year	https://www.resdc.cn/Default.aspx
Watershed vector data		2000, 2010 and 2020 year	https://www.webmap.cn/
Administrative region boundary vector data		2000, 2010 and 2020 year	https://www.webmap.cn/
City Center data		2020 year	https://download.geofabrik.de/
Road quantity data		2020 year	https://download.geofabrik.de/

Land use change is closely related to the natural environment and transportation location. The main spatial variables driving land use change in this paper include natural factors (Digital Elevation Model (DEM), slope, aspect, and soil data) and transportation location factors (distance from city center, town center, railway, expressway, national highway, and provincial highway). Slope and aspect were extracted from the DEM using ArcGIS. The distance data to the city center, town center, railway, expressway, national highway, and provincial highway were further processed using ArcGIS based on the city center data and road network data. The precipitation and evaporation data for the two SSP scenarios in the future periods used in this paper are sourced from the Scenario Model Intercomparison Project (ScenarioMIP) of CMIP6. All raster data have been unified to a resolution of 1000 m × 1000 m, and the coordinate system has been reprojected to Albers_Conic_Equal_Area, with the raster extent maintained consistently.

## 3 Research Methodology

### 3.1 FLUS Model

The FLUS model was developed by Liu et al. (2017) based on the principle of traditional metacellular automata with improvements; the principle is based on the use of artificial neural network algorithms on the basis of the operation of the base period land use data and the data of each driving factor, to estimate the probability of the development of each class in the region, and then the development probability is combined with the domain influence factor, adaptive inertia coefficient, and the cost of conversion to derive the overall probability of conversion of the metacellular. The simulation results are finally derived after a roulette wheel competition mechanism. The principles of neural network‐based suitability probability calculation and adaptive inertia competition mechanism will not be elaborated in detail in this paper, with specific reference to the literature (Liu et al. 2017). In the validation process, we employed the Kappa coefficient, also known as Cohen's Kappa, which is a crucial indicator for measuring the consistency of classification results. It reflects the degree of similarity between the model's simulation results and actual observed data. A Kappa coefficient value closer to 1 indicates a higher level of consistency between the model's simulation results and the actual observed data.

### 3.2 Ecosystem Service Function Assessment Methods

In this paper, the InVEST model was used to quantitatively estimate the HQ, WY, CS, and NDR in Henan Province. Since there have been many related studies describing the specific principles and calculation methods of the above functions, this paper will not elaborate on them in detail, and the specific principles can be found in the user manual of the InVEST model (Sharp et al. 2020).

HQ was assessed using the HQ module in the InVEST model, which reflects the impacts caused by human activities on the environment, and the greater the intensity of human activities, the greater the threat to the habitat. In this paper, we refer to relevant studies (Wang et al. 2022) to select arable land, built‐up land and unused land as stressors, and define woodland, grassland and water as habitats, and refer to the InVEST model user manual and relevant studies (Zhang, Li, et al. 2022) to determine various parameters of the model. The water production service was calculated using the InVEST water production module, which is based on the principle of water balance, subtracting the actual evapotranspiration from the precipitation of each raster to obtain the water production of that raster (Hu et al. 2020). The annual precipitation was adopted as the average value of precipitation from 2000 to 2050, and the parameters of the bio coefficient table were set according to the relevant references (Yang et al. 2020). The *Z* value is a seasonal factor characterizing rainfall, and after several simulations, it is found that the accuracy of the model is higher when the *Z* value is taken as 11.4, and the simulation results are closest to the total water resources of Henan Province in the 2020 Government Water Resources Bulletin, which is 699. 95 billion m^3^, with a relative error of 0.06%. The CS service was calculated using the carbon stock module of the InVEST model, and the average carbon densities of the different land classes were referred to related studies (Yang et al. 2021; Fan et al. 2023). NDR services were calculated using the “NDR” module of the InVEST model, with the parameters required by the model referenced to research settings in similar study areas (Zhang et al. 2022, Wang et al. 2022). The module first determines the number of pollutants retained in each grid in the watershed, and then calculates the total and average pollution loads in the watershed, with the lower the nitrogen output, the higher the water purification service (Cong et al. 2020).

### 3.3 CESs Index

To reflect and quantify the total impacts of multiple ESs, this paper constructs a composite ecosystem services index (CES; Laterra et al. 2012; Wen et al. 2018). In many studies (Pan et al. 2013; Wu et al. 2017), all ESs are given equal weights, but in reality, the importance of different ecosystem service functions is different. Drawing on previous research (Niu et al. 2022), this paper uses the hierarchical analysis method (APH) to construct a CES for comparing the overall level of multiple ESs under different scenarios. The index can spatially reflect the overall condition of urban ecosystems and provide a reference for the government to formulate urban planning policies. The formula for calculating the CES is as follows:

(1) $${\mathrm{CES}}_{j}=\sum_{i=1}^{n}w_{i}.S_{\mathrm{ij}}$$

CES_*j*_ is the comprehensive ecosystem service index in year *j*, *w*_*i*_ is the weight of the *i*th ecosystem service, *S*_*ij*_ is the normalized value of the *i*th ecosystem service in year *j*, and *n* is the number of ecosystem service species.

In this paper, a two‐by‐two comparison of the elements in the impact and indicator layers was used to construct the judgment matrix, and four methods, namely, geometric mean, arithmetic mean, eigenvector, and least squares, were chosen to calculate the weights, and the average of all the calculation methods was taken as the final weights. Table 2 shows the weights of each type of ecosystem service determined by AHP.

**TABLE 2:** The weights of various ES indicators in Henan Province.

Affected layers	The weight of the affected layer	Index layers	The weight of the index layer	Ultimate weight
Regulating services	0.53	Nutrient delivery ratio (nitrogen export)	0.59	0.31
		Carbon storage and sequestration	0.41	0.22
Supplying services	0.24	Annual water yield	1	0.24
Supporting services	0.23	Habitat quality	1	0.23

### 3.4 Geo‐Detectors

In this study, a factor detector was used to detect the extent to which the independent variable X (driver) explains the dependent variable Y (CES) (Wang and Xu 2017), to explore the drivers of changes in ecosystem service trade‐offs and synergistic relationships in terms of both natural and transport locations, which were calculated using the following equation:

(2) $$\begin{array}{c} q=1-\frac{\sum_{h=1}^{L}N_{h}\sigma_{h}^{2}}{N\sigma_{h}^{2}}=1-\frac{\mathrm{SSW}}{\mathrm{SST}} \end{array}$$

(3) $$\begin{array}{c} \mathrm{SSW}=\sum_{h=1}^{L}N_{h}\sigma_{h}^{2},\mathrm{SST}=N\sigma_{h}^{2} \end{array}$$

where *L* is the stratification of the variable, *N*_*h*_ and *N* are the number of cells in stratum *h* and the whole region, $\sigma_{h}^{2}$ and *σ* ^2^ are the variance in stratum *h* and the variance in the region, respectively, and SSW and SST are the sum of the intra‐stratum variance and the total variance in the whole region, respectively. *q* is the explanatory power of the driving factors to the CES, with a range of values of [0,1], and the larger the value of *q*, the greater the degree of the driving factors' contribution to the CES.

### 3.5 Data Analysis

Land use change serves as a critical factor influencing ecological environments and their interactions, while the inverse relationship between land use intensity dynamics and spatial expansion positions it as a pivotal sustainability concern (Erb 2012). Through the application of land use intensity quantification methods—following the methodology of Liu et al. (2019)—we modified the land use intensity classification index (Table 3) to reflect the observed dominance of forestland over grassland coverage in the study area. The specific computational formula can be found in the cited reference.

**TABLE 3:** Relative coefficient of land use type.

Soil type	Farmland	Forestland	Grassland	Waters	Building land	Unused land
Land use intensity	3.0	2.5	2.0	2.0	4.0	1.0

## 4 Results and Analysis

### 4.1 Land Use Change

#### 4.1.1 Land Use Change Simulation

**FIGURE 2:**
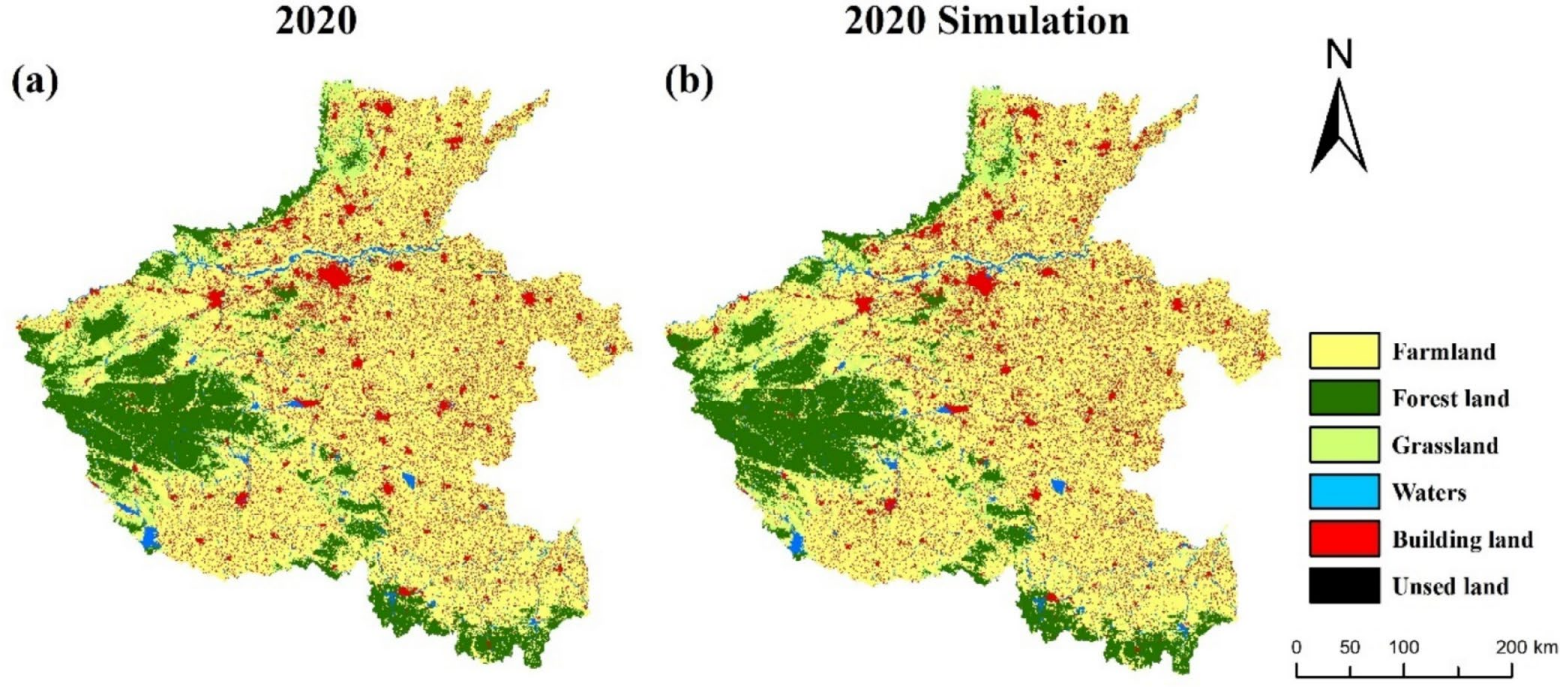
Comparison of 2020 land use simulations. Comparison of actual land use map (a) and simulated land use map (b) in 2020. Comparison of actual land use map (a) and simulated land use map (b) in 2020.

In this study, the Geo SOS‐FLUS model is used to simulate the land use distribution in 2020 based on the current land use map of Henan Province in 2010. After that, the simulated land use distribution in 2020 was compared with the real land use distribution in 2020 (Figure 2). In order to verify the accuracy of the simulation results, the Kappa coefficient was calculated, and the results showed that the average coefficient was 0.94, and the Kappa coefficient was 0.89 (> 0.75), which indicated that the FLUS model has a better simulation ability, and it can provide future land use data for Henan Province for the InVEST model.

In this paper, the global 0.25° × 0.25° LUH2 dataset is used to determine the quantities of each land use type in Henan Province under two scenarios (SSP2‐4.5, SSP5‐8.5) in 2030, 2040, and 2050. Since changes in the number of water bodies are not predicted in the LUH2 dataset, this paper assumes that the number of water bodies under different SSP‐RCP scenarios in 2050 remains the same as the number of water bodies in 2020. In addition, the area of five types of land in the LUH2 dataset, including cropland, forest land, grassland, construction land, and unutilized land, is significantly different from its actual area. In order to avoid the errors caused by the future land use simulation, this paper uses the FLUS model to simulate the future land use area of each type of land in LUH2 based on the actual land use data of the study area in 2020. The calibration process is as follows: first, replace the area of corresponding land types in LUH2 with the actual area of each land use type in 2020; then, calculate the proportion of changes in the five land use types from 2020 to 2030, 2040, and 2050 under different scenarios; finally, multiply the actual area of each land use type in 2020 by the proportion of changes in LUH2 to obtain the proportion of changes in 2030, 2040, and 2050, and finally, multiply the actual area of each land use type in 2020 by the proportion of changes in LUH2. Finally, by multiplying the actual area of each land use type in 2020 by the proportion of change of each land use type in LUH2, the predicted area of each land use type in 2030, 2040, and 2050 can be obtained. Finally, the demand for each land use type under the two scenarios (SSP2‐4.5 and SSP5‐8.5) in Henan Province in 2030, 2040, and 2050 is shown in Table 4.

**TABLE 4:** Demand for land use types under different SSP scenarios in Henan Province (km^2^).

Land use type	SSP2‐4.5			SSP5‐8.5		
	2030	2040	2050	2030	2040	2050
Farmland	105,172	105,777	106,299	105,919	106,262	106,941
Forestland	27,071	26,780	26,708	26,194	26,153	25,371
Grassland	8002	7461	6926	8186	7697	7631
Waters	4324	4324	4324	4324	4324	4324
Building land	22,398	22,625	22,710	22,344	22,531	22,700
Unused land	33	33	33	33	33	33

#### 4.1.2 Land Use Changes From 2000 to 2020

Based on the land use data of Henan Province in 2000, 2010, and 2020, using ArcGIS software to analyze to get the transfer matrix of land use types in Henan Province in 2000–2010 and 2010–2020 (Tables 5 and 6), according to the data of the transfer matrix, the change in the area of six land use types in Henan Province can be found.

**TABLE 5:** Land use transfer matrix from 2000 to 2010 (km^2^).

2010	Farmland	Forestland	Grassland	Waters	Building land	Unused land	Total
2000							
Farmland	92180.03	2690.12	2062.62	1656.68	10678.75	7.56	109275.76
Forestland	2594.09	23009.68	1006.80	216.10	274.05	4.02	27104.75
Grassland	2228.06	1194.07	5674.90	164.10	286.96	8.32	9556.42
Waters	1289.59	143.94	88.35	1788.10	209.34	1.40	3520.72
Building land	9242.05	82.42	98.97	182.52	7840.87	0.21	17447.04
Unused land	46.99	16.12	3.42	19.49	3.68	5.62	95.32
Total	107580.82	27136.36	8935.06	4026.99	19293.65	27.13	167000.00

**TABLE 6:** Land use transfer matrix from 2010 to 2020 (km^2^).

2020	Farmland	Forestland	Grassland	Waters	Building land	Unused land	Total
2010							
Farmland	103323.20	597.86	93.76	352.87	3078.03	0.00	107445.72
Forestland	499.06	26435.97	262.13	48.39	47.39	10.08	27303.02
Grassland	95.78	195.59	8570.69	31.25	38.31	0.00	8931.63
Waters	116.95	14.11	3.02	3878.54	28.23	1.01	4041.87
Building land	413.36	11.09	6.05	13.11	18805.92	1.01	19250.54
Unused land	2.02	3.02	1.01	0.00	0.00	21.17	27.22
Total	104450.36	27257.65	8936.67	4324.16	21997.88	33.27	167000.00

Through the analysis, it was found that from 2000 to 2010, the area of forest land, water area and construction land in Henan Province increased by 1846.61 km^2^ (1.11%), 506.27 km^2^ (0.30%) and 31.61 km^2^ (0.02%), while the area of arable land, grassland and unutilized land decreased by 1694.94 km^2^ (1.01%), 621.35 km^2^ (0.37%) and 68.19 km^2^ (0.04%), respectively, 621.35 km^2^ (0.37%) and 68.19 km^2^ (0.04%) respectively. From 2010 to 2020, the areas of grassland, water, construction land and unutilized land in Henan Province will increase by 5.04 km^2^ (0.003%), 282.30 km^2^ (0.17%), 2747.34 km^2^ (1.65%) and 6.05 km^2^ (0.004%) respectively, and the areas of arable land and forest land will decrease by 2995.36 km^2^ (1.79%) and 45.37 km^2^ (0.03%) respectively. As can be seen from Tables 5 and 6, the reduced cropland area has been primarily converted to construction land, which aligns with the actual urban expansion observed in Henan Province over the past two decades. The increased forestland area from 2000 to 2010 mainly originated from conversions of cropland and grassland, primarily attributed to the implementation of the “Grain for Green Program” ecological restoration program. Conversely, the decrease in forestland area from 2010 to 2020 resulted from the “returning forestland to cropland” policy adjustments aimed at ensuring food security. Changes in unutilized land are complex and it is not possible to determine the main sources. In conclusion, from 2000 to 2020, the area of waters and construction land in Henan Province increases, respectively by 788.56 km^2^ (0.47%) and 4593.95 km^2^ (2.75%), and the area of cultivated land, forest land, grassland and unutilized land decreases, respectively by 4690.30 km^2^ (2.81%), 13.76 km^2^ (0.01%), 616.31 km^2^ (0.37%), and 62.14 km^2^ (0.04%) respectively.

### 4.2 Changes in ESs Under Different Scenarios in Henan Province, 2000–2050

#### 4.2.1 Changes in the Supply of ESs

According to Table 7, water production, HQ, carbon stock, and nitrogen output in Henan Province in 2000 were 70.04 × 10^9^ m^3^, 0.376, 16.06 × 10^8^ t, and 27.65 × 10^3^ t, respectively. In 2010, water production, carbon stock, and nitrogen output decreased by 18.93 × 10^9^ m^3^, 8.71 × 10^6^ t, and 545.35 t, respectively, while HQ increased from 0.376 to 0.380. In 2020, carbon stocks decreased by 10.69 × 10^6^ t, water production and nitrogen output increased by 18.01 × 10^9^ m^3^ and 58.38 t, and HQ increased from 0.380 to 0.396.

**TABLE 7:** Ecosystem service supply and its percentage change from 2000 to 2050.

Indicators	2000	2010	2020	SSP2‐4.5			SSP5‐8.5		
				2030	2040	2050	2030	2040	2050
Annual water yield/(10^9^ m^3^)		51.11	69.12	62.68	43.41	39.80	74.72	80.52	86.59
Change rate/%		−27.02	35.23	−9.31	−30.75	−8.30	8.10	7.77	7.53
Habitat quality	0.376	0.380	0.396	0.375	0.371	0.370	0.371	0.370	0.366
Change rate/%		1.06	4.21	−5.30	−1.07	−0.27	−6.31	−0.27	−1.08
Carbon storage/(10^8^t)	16.06	15.97	15.86	15.83	15.81	15.80	15.80	15.79	15.76
Change rate/%		−0.54	−0.67	−0.19	−0.15	−0.07	−0.37	−0.08	−0.22
Nitrogen export/(10^3^t)	27.65	27.10	27.16	27.15	27.75	27.83	27.69	27.44	28.11
Change rate/%		−1.97	0.22	−0.02	2.20	0.28	1.97	−0.92	2.43

Under the SSP2‐4.5 scenario, water production, HQ, and CS decrease each year from 2030 to 2050, and nitrogen export decreases and then increases. Under the SSP2‐4.5 scenario, water production decreased by 6.44 × 10^9^, 19.28 × 10^9^, and 3.60 × 10^9^ m^3^ from 2030 to 2050, HQ decreased by 0.021, 0.004, and 0.001 from 2030 to 2050, CS decreased by 2.95 × 10^6^, 2.36 × 10^6^, and 1.13 × 10^6^ t from 2030 to 2050, and nitrogen output decreased by 4.96 t in 2030, increased by 597.35 t in 2040, and increased by 77.27 t in 2050. In summary, 2030–2050 water production, HQ, and CS decreased by 29.31 × 10^9^ m^3^, 0.026, and 6.44 × 10^6^ t, and nitrogen output increased by 669.63 t.

Under the SSP5‐8.5 scenario, from 2030 to 2050, HQ and carbon stocks decrease every year, water production increases every year, and nitrogen output increases, then decreases, then increases again. Under the SSP5‐8.5 scenario, HQ decreases by 0.025, 0.001, and 0.004 from 2030 to 2050, and carbon stocks decrease by 5.84 × 10^6^, 1.25 × 10^6^, and 3.48 × 10^6^ t from 2030 to 2050, and water production from 2030 to 2050 increased by 5.60 × 10^9^, 5.81 × 10^9^, and 6.07 × 10^9^ m^3^ from 2030 to 2050, and nitrogen output increased by 536.14 t in 2030, decreased by 255.69 t in 2040, and increased by 667.80 t in 2050. In summary, 2030–2050 HQ and carbon stocks decreased by 0.030 and 10.57 × 10^6^ t, while water production and nitrogen output increased by 17.47 × 10^9^ m^3^ and 948.24 t.

#### 4.2.2 Changes in Spatial Distribution Patterns of ESs

**FIGURE 3:**
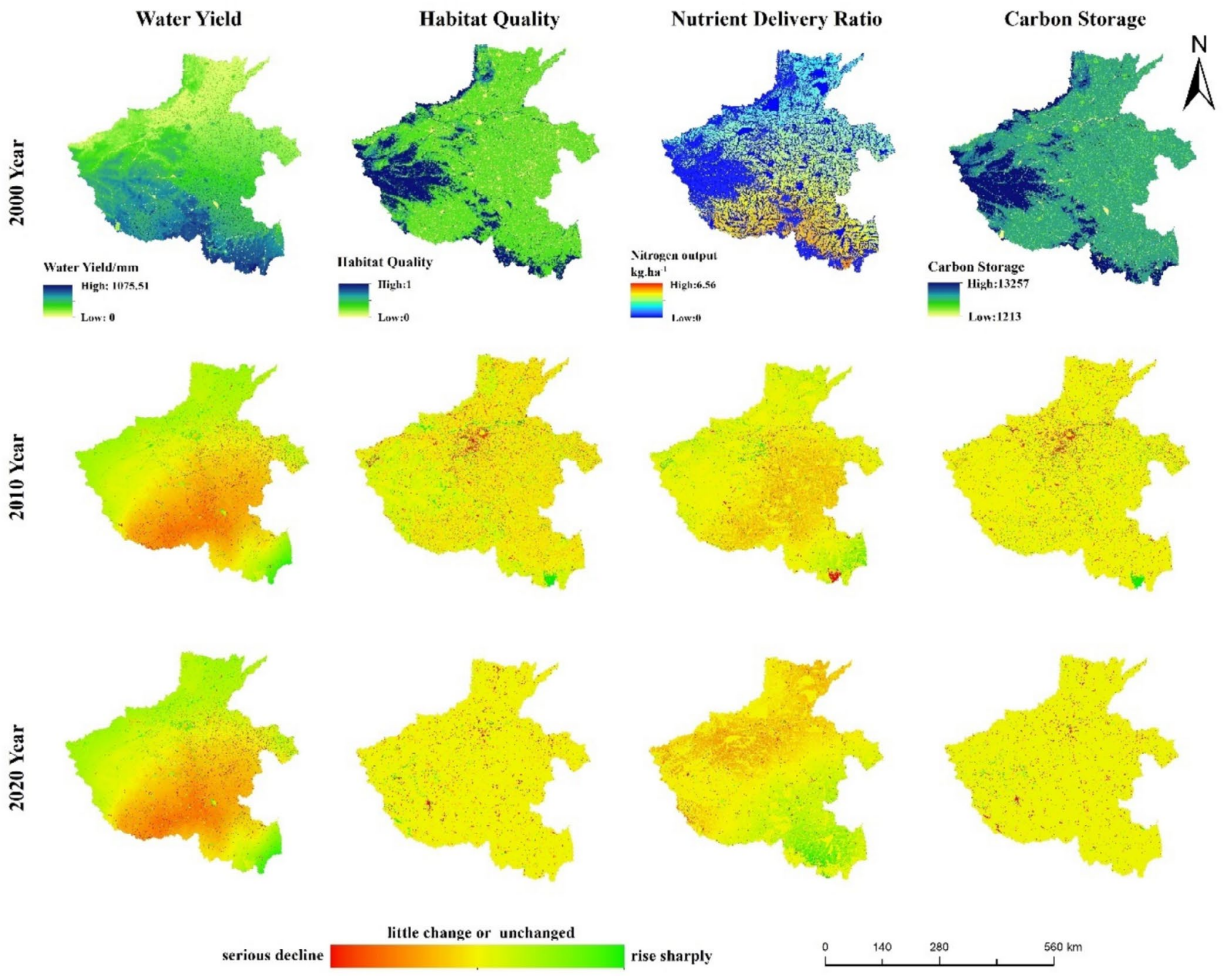
Spatial distribution of ES in 2000 and spatial distribution of changes in ES in 2010–2020.

**FIGURE 4:**
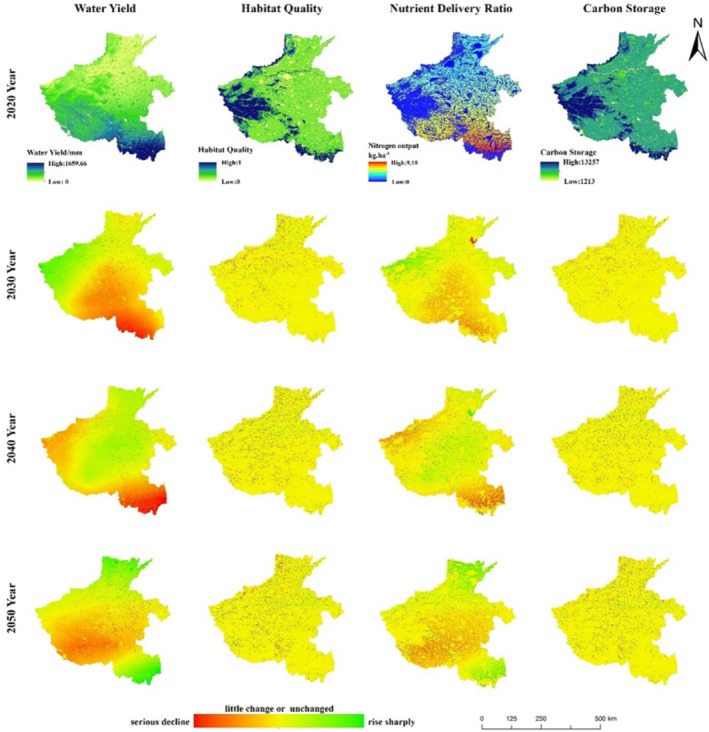
Spatial distribution of ES in 2020 and spatial distribution of changes in ES under the SSP2‐4.5 scenario.

**FIGURE 5:**
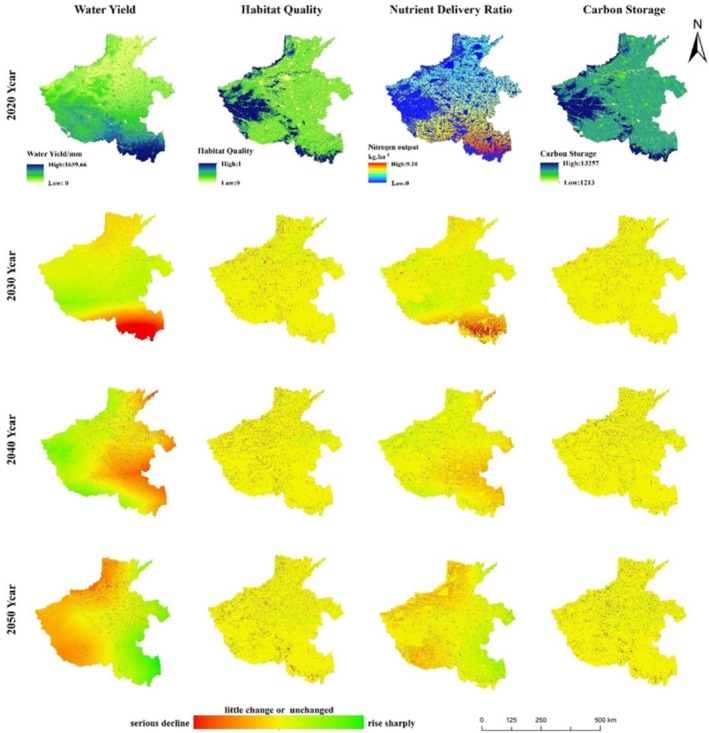
Spatial distribution of ES in 2020 and spatial distribution of changes in ES under the SSP5‐8.5 scenario.

This study maps the spatial distribution pattern of ESs in 2000 and for 2010 and 2020 (Figure 3), and the spatial distribution pattern of ESs in 2020 and for changes from 2030 to 2050 under the two future scenarios (Figures 4 and 5). The results of the study showed that the spatial distribution patterns of HQ and CS in Henan Province were relatively similar, with high values distributed in the western, northwestern, and southwestern regions with high vegetation cover, low values distributed in densely populated urban areas, and CS in cropland at an intermediate level, and the high‐value areas of the two ESs were all regions with high vegetation cover. The spatial distribution pattern of water output is related to precipitation, with high values mainly distributed in the southern and western regions, and low values mainly distributed in the plains in the north. The low values of nitrogen output are mainly distributed in the western, northwestern, and southwestern mountainous areas, which are basically the same as the distribution of woodland and grassland, while the high values are mainly distributed in the southwestern and southeastern plains areas where cultivated land is concentrated.

From 2000 to 2020, HQ and carbon stocks were most severely degraded around urban centers, while degradation of varying degrees also existed around many mountain ranges in Henan Province, especially along Song mountain. This was due to the massive expansion of urban land use and rural settlements encroaching on cropland, woodland, and grassland, resulting in a significant decline in HQ and carbon stock. Water production and nitrogen output, on the other hand, showed an increasing trend in the southeast and a decreasing trend in the center and north, both of which were more related to the distribution of precipitation. Continued degradation of HQ and carbon stocks under the SSP2‐4.5 scenario 2030–2050, with water production decreasing each year and nitrogen output decreasing and then increasing, with most of the decrease occurring around cropland. In 2030–2050 under the SSP5‐8.5 scenario, the degradation of HQ, carbon stocks, and water purification increased compared to the SSP2‐4.5 scenario, except for water production, which increased every year.

### 4.3 Changes in CES Index Under Different Scenarios, 2000–2050

#### 4.3.1 Analysis of Factors Influencing the CES in Henan Province

**FIGURE 6:**
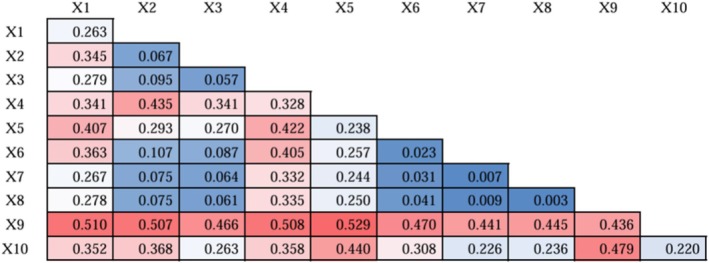
Geodetector two‐factor detection weighted hotspot map.

Geoprobes were used to explore the explanatory power of each factor on the differences in the spatial distribution of CES, and the results of single‐factor probes are shown in Table 8, and the results of interactive probes of each factor are shown in Figure 6. Where X1, X2, …, X10 represent the gross domestic product (GDP), normalized difference vegetation index (NDVI), night light data, population density, temperature, oxygen content of soil root, optimum soil tilth, soil salt salinity, soil type, and land use intensity as the 10 driving factors.

**TABLE 8:** Geodetector single‐factor detection weighting results.

Impact factor	X1	X2	X3	X4	X5	X6	X7	X8	X9	X10
*q* statistic	0.263	0.067	0.057	0.328	0.238	0.023	0.007	0.003	0.436	0.220
*p* value	0.000	0.000	0.000	0.000	0.000	0.000	0.000	0.000	0.000	0.000

As indicated in Table 8, the p‐values for all factors are 0, suggesting that each factor has a significant explanatory power. The *q*‐value for soil type is the highest at 0.436, indicating that soil type has the strongest explanatory power for the spatial distribution differences of CES. Optimum soil tilth and soil salt salinity have the weakest explanatory power for the spatial distribution differences of CES, with *q*‐values of 0.007 and 0.003, respectively. The influence of each factor, in descending order, is as follows: soil type > population density > GDP > temperature > land use intensity > NDVI > Night light data > Oxygen content of soil root > optimum soil tilth > soil salt salinity.

Figure 6 reveals that after interacting with each other, all influencing factors significantly enhance the impact on the spatial distribution differences of CES in the study area. The interaction between factors shows effects of bilinear enhancement and nonlinear enhancement, without any instances of mutual independence or weakening. Notably, the interaction between soil type and temperature is the strongest, followed by the interaction between soil type and GDP. The maximum value of soil type∩Temperature (aspect, *q* = 0.529) indicates that the interaction of soil type and Temperature exhibits stronger explanatory power. Considering the results of single‐factor detection, the spatial heterogeneity of CES in Henan Province is the result of the interaction and enhancement of multiple factors, rather than the result of the independent action of a single factor. Combining the results of single‐factor detection, the spatial heterogeneity of CES in Henan Province is the outcome of the interaction and enhancement of multiple factors, rather than the result of the independent action of a single factor.

#### 4.3.2 Changes In the Average Value of CES in Henan Province

The average value of CES can reflect the overall supply status of ESs, and the average values of CES in Henan Province in 2000, 2010, and 2020 were 0.569, 0.544 (decreased by 4.53%) and 0.553 (increased by 1.65%), respectively, which showed a decreasing and then increasing trend. The 2030, 2040, and 2050 CES averages under the SSP2‐4.5 scenario are 0.543 (down 1.47%), 0.533 (down 1.82%), and 0.529 (down 0.74%), respectively, showing a continuing downward trend. The 2030, 2040, and 2050 CES averages under the SSP5‐8.5 scenario are 0.551 (up by 4.13%), 0.563 (up by 2.23%) and 0.550 (down by 2.33%), showing an increasing and then decreasing trend. From the magnitude of the mean CES values for 2030–2050 under the two different scenarios, it can be judged that the mean CES values are larger in the SSP5‐8.5 scenario. This indicates that the SSP5‐8.5 scenario is more suitable for the future development of Henan Province than the SSP2‐4.5 scenario.

**FIGURE 7:**
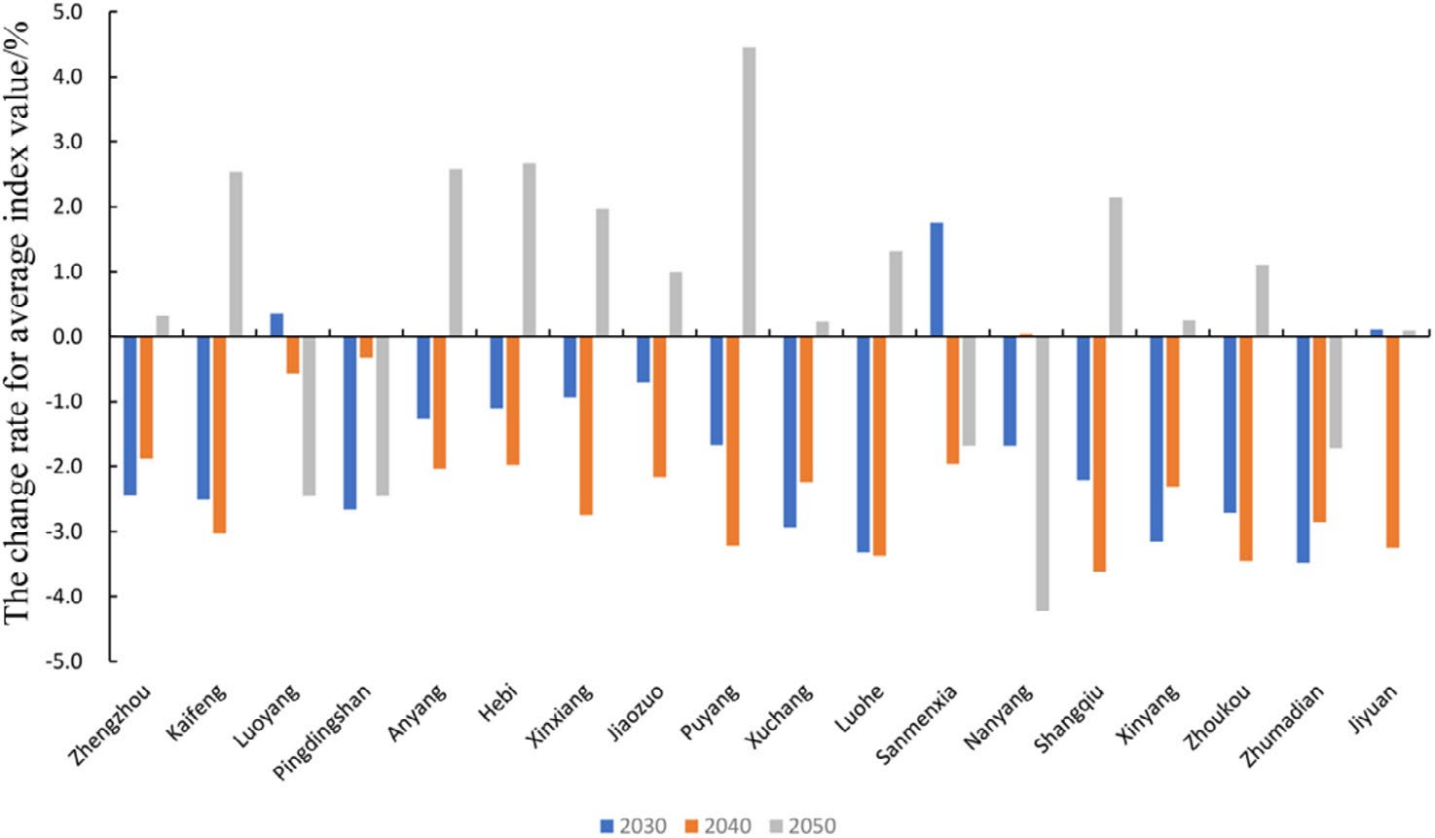
Percentage change of CES mean across cities in Henan Province under the SSP2‐4.5 scenario.

**FIGURE 8:**
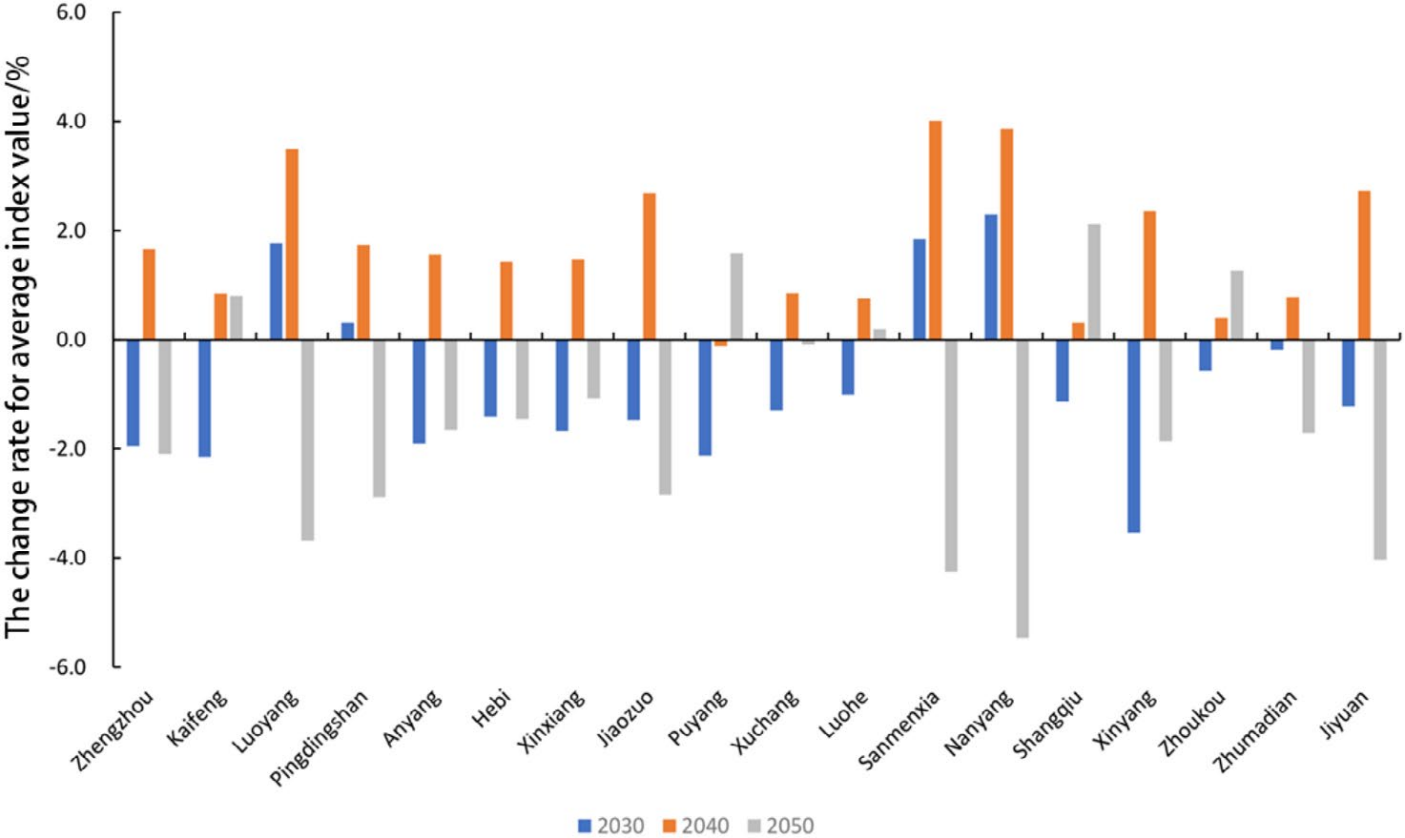
Percentage change of CES mean across cities in Henan Province under SSP5‐8.5 scenario.

The average annual changes in CES from 2000 to 2050 in each city of Henan Province are shown in Table 9, while the percentage changes in the average CES in each city under different scenarios are shown in Figures 7 and 8. The above graphs and tables show that Luoyang, Sanmenxia, Nanyang, Xinyang, and Jiyuan had higher CES mean values, and woodland and grassland were mainly distributed in these cities. On the other hand, Zhengzhou, Kaifeng, Puyang, Xuchang, Luohe, Shangqiu, and Zhoukou had lower forest and grassland cover and lower CES mean values. In 2030–2050 under the SSP2‐4.5 scenario, except for the seven cities of Luoyang, Pingdingshan, Sanmenxia, Nanyang, Xinyang, Zhumadian, and Jiyuan, the CES averages of the rest of the cities in the last three decades generally show a downward and then upward trend, with Puyang City showing the greatest increase. In 2030–2050 under the SSP5‐8.5 scenario, only Luoyang, Pingdingshan, Sanmenxia, and Nanyang CES averages coincide with the overall changes in Henan Province, while Kaifeng, Luohe, Shangqiu, and Zhoukou CES averages first decrease and then increase, and CES averages in all other municipalities first decrease, then increase, and then decrease. Sanmenxia and Nanyang have the largest decreases among all the cities.

**TABLE 9:** Changes in the average value of CES across municipalities in Henan Province, 2000–2050.

City	2000	2010	2020	SSP2‐4.5			SSP5‐8.5		
				2030	2040	2050	2030	2040	2050
Zhengzhou	0.532	0.490	0.495	0.482	0.473	0.475	0.485	0.493	0.483
Kaifeng	0.477	0.457	0.468	0.457	0.443	0.454	0.458	0.462	0.466
Luoyang	0.652	0.633	0.632	0.634	0.631	0.615	0.643	0.666	0.641
Pingdingshan	0.585	0.554	0.557	0.542	0.541	0.527	0.559	0.569	0.552
Anyang	0.518	0.507	0.514	0.508	0.497	0.510	0.504	0.512	0.504
Hebi	0.514	0.506	0.511	0.505	0.495	0.509	0.504	0.511	0.504
Xinxiang	0.504	0.493	0.500	0.495	0.482	0.491	0.492	0.499	0.493
Jiaozuo	0.532	0.515	0.521	0.517	0.506	0.511	0.513	0.527	0.512
Puyang	0.469	0.456	0.466	0.458	0.443	0.463	0.456	0.455	0.462
Xuchang	0.505	0.475	0.485	0.471	0.460	0.461	0.479	0.483	0.482
Luohe	0.486	0.454	0.470	0.455	0.439	0.445	0.465	0.469	0.470
Sanmenxia	0.672	0.661	0.655	0.666	0.653	0.642	0.667	0.693	0.664
Nanyang	0.647	0.609	0.612	0.602	0.602	0.577	0.626	0.650	0.615
Shangqiu	0.476	0.457	0.469	0.459	0.442	0.452	0.464	0.465	0.475
Xinyang	0.620	0.600	0.624	0.604	0.590	0.592	0.602	0.616	0.605
Zhoukou	0.486	0.456	0.472	0.460	0.444	0.449	0.470	0.472	0.478
Zhumadian	0.552	0.509	0.528	0.510	0.495	0.487	0.527	0.532	0.522
Jiyuan	0.615	0.606	0.608	0.609	0.589	0.590	0.601	0.617	0.592

#### 4.3.3 Characteristics of Spatial Changes of Comprehensive Ecosystem Index in Henan Province

**FIGURE 9:**
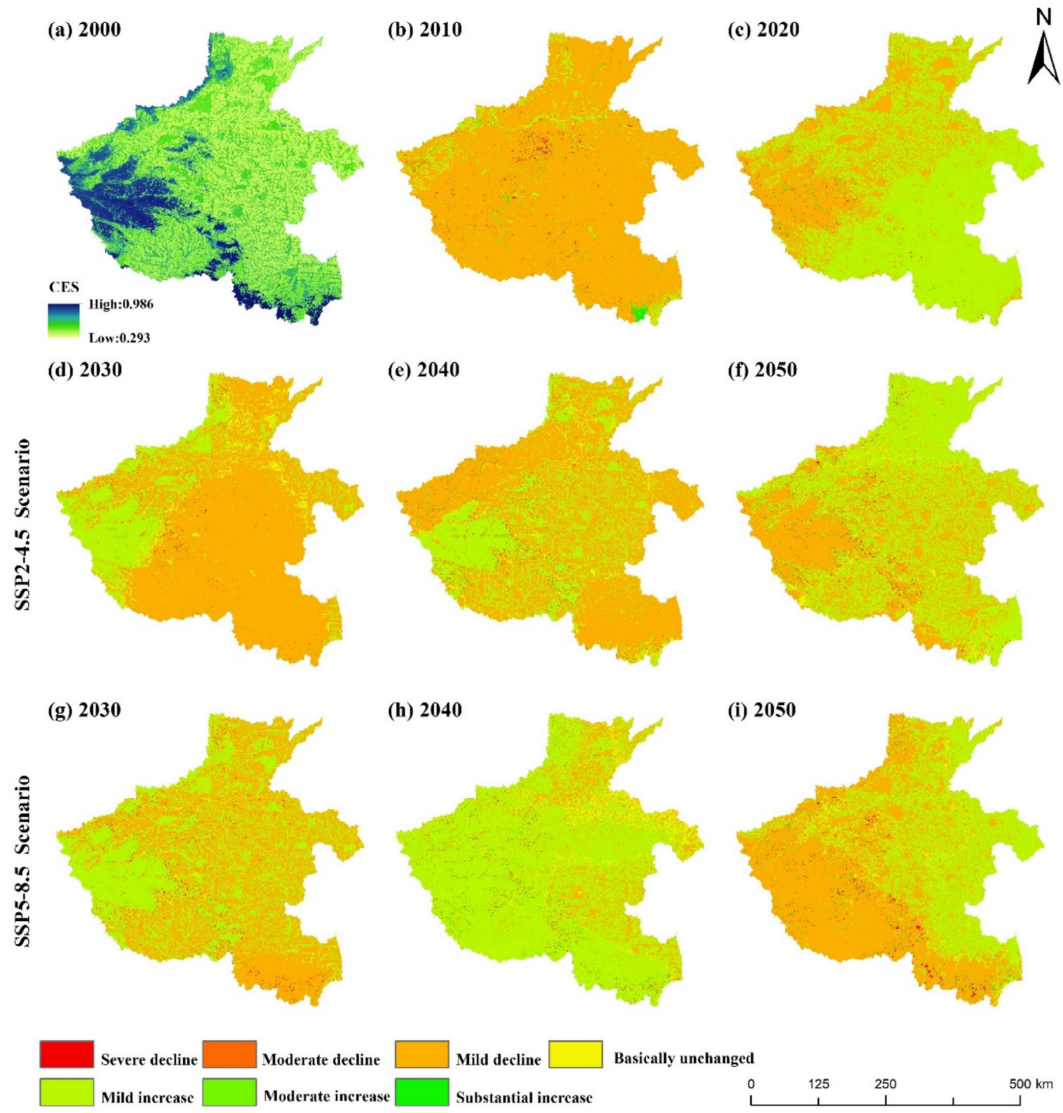
Patterns of spatial distribution of CES in 2000 and the spatial distribution of CES changes under different scenarios in 2010–2050.

The results of the study (Figure 9) show that the CES of urban land, rural settlements, and other construction land in the urban centers of each city is low, while the CES of forest land and grassland in the Furniu Mountain and Dabie Mountain regions is high. In order to more clearly reflect the spatial changes of CES under different scenarios in Henan Province, the CES distribution maps before and after the two periods were subjected to raster subtraction, and the change values between ±0.001 were defined as basically unchanged areas (Leh et al. 2013), and the change values were classified into seven levels by combining with the natural breakpoint method: severe decline (< −0.30), moderate decline (−0.30 to 0.15), mild decline (−0.15 to 0.001), essentially unchanged (−0.001 to 0.001), mildly increasing (0.001 to 0.12), moderately increasing (0.12 to 0.24), and substantially increasing (> 0.24).

**FIGURE 10:**
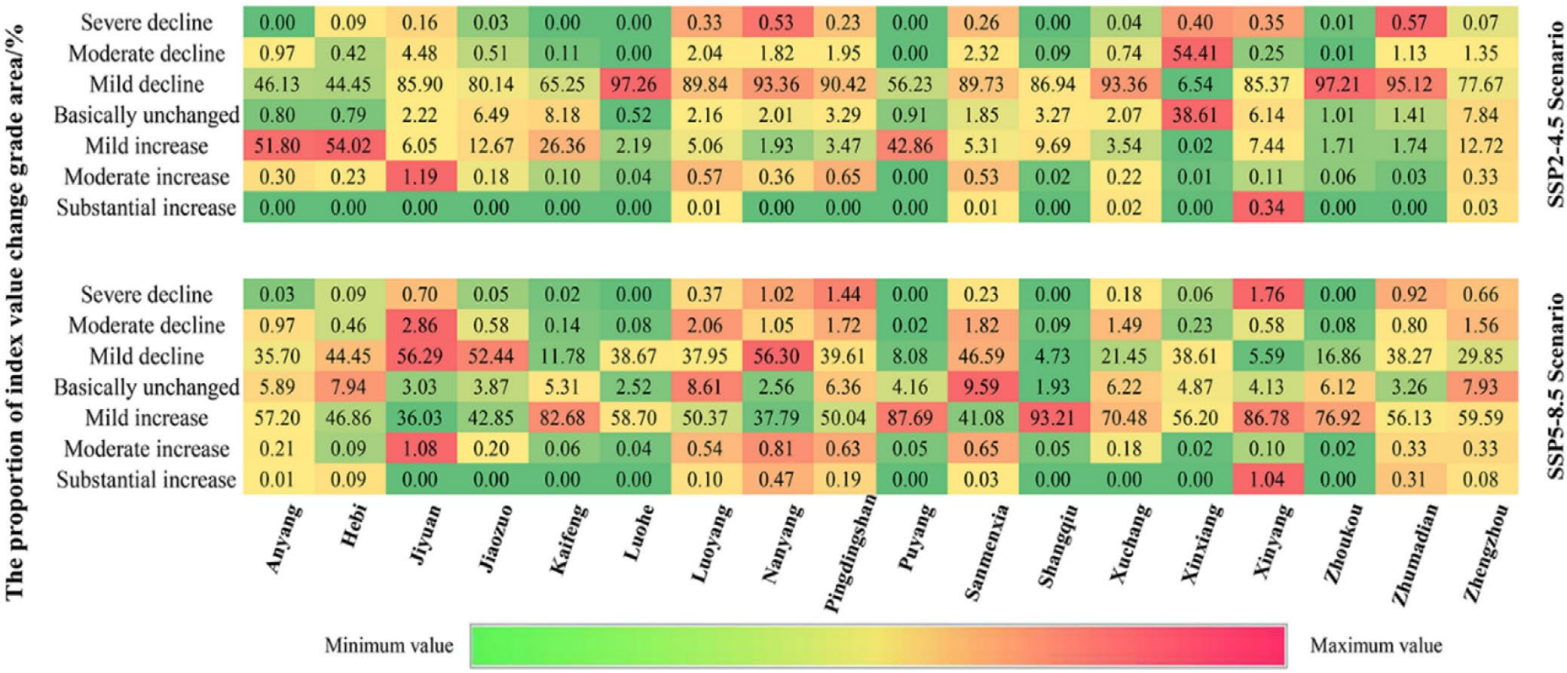
Percentage change in average CES across municipalities in Henan Province, 2030–2050.

The overall 2030–2050 share of area change in CES class for each city is shown in Figure 10. Under the SSP2‐4.5 scenario, Zhumadian City has the highest ratio of the area of severely declining CES regions, accounting for 0.57% of the overall CES change in Zhumadian City. Xinxiang City has the highest ratio of the area of CES moderately declining areas, accounting for 54.41% of the overall CES change in Xinxiang City. Luohe and Zhoukou CES mildly declining areas accounted for more than 97% of the ratio value. Hebi City has the highest percentage of CES mildly increasing area, accounting for 54.02% of the overall CES change in Hebi City. Jiyuan City has the highest percentage of CES moderate increase area, accounting for 1.19% of the overall CES change in Jiyuan City. Xinyang City has the highest percentage of CES substantially increasing area, accounting for 0.34% of the overall CES change in Xinyang City. In 2030–2050 under the SSP2‐4.5 scenario, the overall CES decreasing area is the largest, accounting for 85.38% of the total area, and the area of the CES rising area accounts for 11.40% of the total area, and the CES rising area is mainly located in the areas of Anyang, Hebi, and Puyang City, and the main reason for the rise is the expansion of woodland and grassland.

Figure 10 shows that under the SSP5‐8.5 scenario, Xinyang City has the highest ratio of the area of severely declining CES regions, accounting for 1.76% of the overall CES change in Xinyang City. Jiyuan City has the highest percentage of CES moderately declining area, accounting for 2.86% of the overall CES change in Jiyuan City. Nanyang City has the highest percentage of CES mildly decreasing area, accounting for 56.30% of the overall CES change in Nanyang City. Shangqiu City has the highest percentage of CES mildly increasing area, accounting for 93.21% of the overall CES change in Shangqiu City. Jiyuan City has the highest percentage of CES moderate increase area, accounting for 1.08% of the overall CES change in Jiyuan City. Xinyang City has the highest percentage of CES substantially increasing area, accounting for 1.04% of the overall CES change in Xinyang City. Under the SSP5‐8.5 scenario, the overall CES increases the most, accounting for 61.28% of the total area, and the CES decreases the most, accounting for 33.65% of the total area. The CES decreases are mainly in the areas with more forest and grassland resources, and the main reason for the decreases is the expansion of urban land and rural settlements.

## 5 Discussion

### 5.1 Accuracy of Simulation Results and Comparison

In this study, the InVEST model was used to calculate four ESs for Henan Province: annual WY, HQ, CS, and nitrogen export. Since the model validation for WY in Henan Province has already been described in Section 3.3, it will not be repeated here. Additionally, the results of HQ are greatly influenced by threat sources, and different researchers may use different threat source data. Therefore, we compare the results of CS and nitrogen export with existing research findings to further validate the accuracy of the model.

In previous studies, Fan et al. (2023) reported total CS of 59.42 × 10^8^, 58.62 × 10^8^, and 57.54 × 10^8^ t for the years 2000, 2010, and 2020, respectively; Wang et al. (2024) found total CS of 21.95 × 10^8^ and 21.07 × 10^8^ t for 2010 and 2020. Tao et al. (2021) used the export coefficient method to simulate non‐point source pollution in the Yellow River basin, obtaining total nitrogen exports of 9.93 × 10^4^ and 4.08 × 10^4^ t/a for Henan Province in 2006 and 2017, respectively. Comparing these results with the present study, the estimated values of CS and nitrogen export are consistent in order of magnitude but differ in specific values, which may be attributed to differences in data sources, study periods, and simulation precision among the various models used.

### 5.2 Impacts of Land Use Change on ESs

**FIGURE 11:**
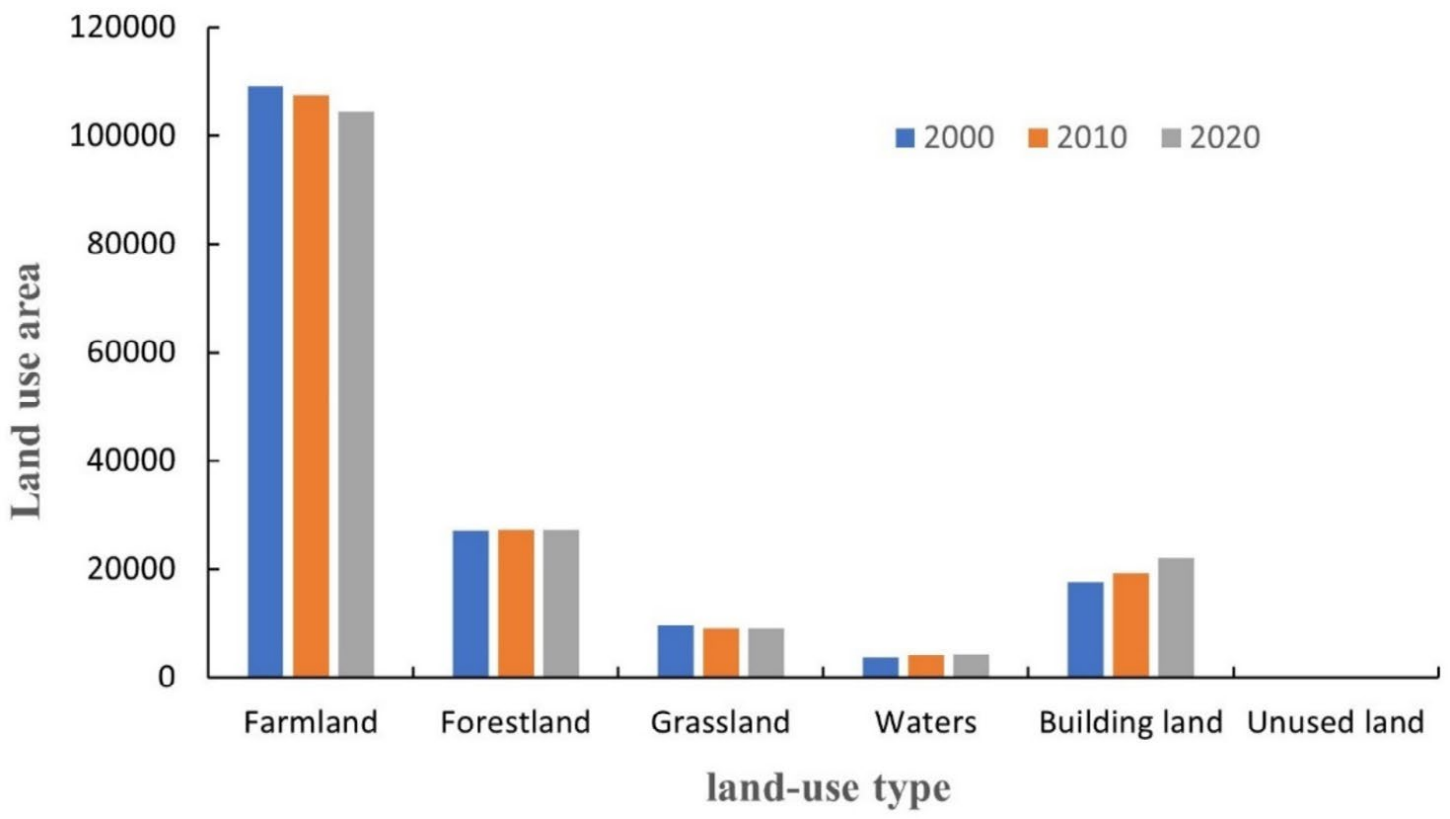
Histogram of land use area change 2000–2020.

Based on the map of land use type changes in Henan Province from 2000 to 2020 (Figure 11), combined with the changes in ecological service functions and CES in Henan Province from 2000 to 2020 summarized in the previous analysis, it can be concluded that the increase in construction land use will lead to the destruction of carbon stocks and HQ, which will affect the decline of CES (Li, Ye, et al. 2021, Li, Ling, et al. 2021). Meanwhile, it was found that the increase in the area of built‐up land did not necessarily reduce the value of ESs, and although the area of built‐up land in Henan Province increased year by year from 2000 to 2020, the average value of CES in 2020 increased by 1.65% compared with that in 2010. This may be due to the effect of the increase in grassland area in 2020 or the effect of other factors on the ecosystem service function, which needs to be analyzed in further research.

Meanwhile, overlaying the CES changes with land use changes and ecosystem service function changes, it was found that the severe decline of CES in the SSP2‐4.5 scenario occurred mainly in the areas where forest land and arable land were encroached upon by the construction land, which caused the simultaneous decline of HQ, CS, water production, and water purification capacity, and the moderate decline of CES occurred mainly in the areas where grassland was encroached upon by the construction land, which caused the simultaneous decline of water production, HQ, CS, and water purification capacity. The moderate decline in CES occurred mainly in areas where grassland was encroached upon by construction land, causing a simultaneous decline in water quantity, HQ, CS, and water purification capacity, while the mild decline in CES accounted for the largest proportion of the declining areas, mainly due to the decline in HQ of the encroached cropland around the cropland encroached upon by the construction land.

Under the SSP5‐8.5 scenario, there are very few areas of severe and moderate decline in CES, and the areas of mild decline are dominated by simultaneous declines in WY, CS, and HQ, with the largest area of cropland encroached by rural settlements. The area of CES rise is the largest, with mild rise dominated by increases in HQ and WY, and the conversion of land type is mainly from grassland to woodland. The areas of moderate rise are the second largest, with the largest area of moderate rise mainly due to the conversion of cropland to woodland. The area of moderate rise is the second largest, mainly due to the conversion of grassland to woodland. The second largest area of CES is the area of moderate increase, which mainly occurs in the area of returning farmland to forest, and the area of large increase in CES is dominated by the increase in HQ and carbon stock service, which mainly occurs in the area of converting constructed land to forest land and grassland.

### 5.3 Causes and Recommendations

The present study investigates the spatiotemporal changes in ESs from the perspective of land use, and the findings align with existing research. CS, heavily influenced by land use, is primarily affected by changes in forest, cropland, and grassland, consistent with Zhang et al.'s (2024) research. The significant expansion of construction land, leading to the degradation of HQ, parallels Zhao et al.'s (2024) findings. Although Anhui and Henan are different regions, both are major agricultural provinces in China with similar patterns of urban expansion, resulting in similar research outcomes. WY and nitrogen export are influenced by land use type changes, but according to Hu et al. (2022) and Li et al. (2020), both are more significantly affected by precipitation. The change in CES under the SSP2‐4.5 scenario may be due to the dual impact of decreasing annual precipitation and land use changes. The change in CES under the SSP5‐8.5 scenario could be attributed to the conversion of cropland, forest, and grassland into construction land, causing losses in CS and HQ, thereby leading to changes in CES.

In response to the changing ecosystem service functions in different cities of Henan Province under various scenarios, we propose the following recommendations. Under the SSP2‐4.5 scenario, where the overall average CES in Henan Province shows a declining trend, it is crucial to be vigilant against the risks associated with the deterioration of ecosystem functions. Appropriate policies should be formulated for different cities based on local conditions. For cities like Luoyang, Pingdingshan, Sanmenxia, Nanyang, Xinyang, Zhumadian, and Jiyuan, where CES is expected to continue declining, special attention should be given to the impacts of climate change on land use structures. Efforts should be made to protect and restore ecologically vulnerable areas. For cities experiencing fluctuations in CES, it is important to focus on the coordinated development of ecological functions across regions and formulate comprehensive ecological stability policies to ensure the healthy and stable development of regional ecosystems.

Under the SSP5‐8.5 scenario, for cities like Kaifeng, Luohe, Shangqiu, and Zhoukou, where CES changes are inconsistent with the overall CES trend of Henan Province, it is necessary to be alert to sudden ecological disasters caused by unexpected factors. Emergency response measures should be developed to prevent the destruction of ecosystem functions. For other cities in this scenario, it is recommended to implement key ecological protection and restoration projects. Priority should be given to the ecological restoration of degraded wetlands and areas with sparse vegetation, enhancing their water purification and carbon sequestration functions. Strict control should be exercised over agricultural and industrial pollution sources to reduce the entry of pollutants into wetlands, ensuring the effectiveness of ecological restoration.

### 5.4 Shortcomings and Prospects

The present study has some limitations. First, in terms of land use prediction, this study is based on the findings of previous research, which may introduce some degree of lag (Schirpke et al. 2020). Second, while this research investigates the impact of land use change on multiple ESs, data limitations restricted the selection to only four ESs. Additionally, the influence of land use change on WY and nitrogen export is confounded by the effects of precipitation, making it difficult to isolate the impact of land use change on these two variables (Ding et al. 2021). Future research aims to address these limitations by incorporating regional‐specific conditions and studying other ESs such as food production, soil and water conservation, and cultural services (Cybèle et al. 2024) in Henan Province. This will allow for a more comprehensive and integrated assessment of the effects of land use change on a diverse range of ESs.

## 6 Conclusions

In this paper, the land use types of Henan Province in 2030, 2040, and 2050 under two different climate scenarios were simulated by using the FLUS model combined with the LUH2 dataset. After that, the ecosystem service functions of four modules, namely water production, CS, HQ, and water purification, were calculated using the InVEST model, and the CES was constructed. Finally, the temporal and spatial changes of each ecosystem service function and CES were analyzed, and the conclusions are as follows:

1. The Kappa coefficient of the FLUS model is 0.87, indicating that the FLUS model is suitable for land use simulation in the study area. The simulation results showed that under the SSP2‐4.5 scenario, the expansion area of construction land was the most significant. Under the SSP5‐8.5 scenario, the area of cultivated land increased the most, by 2491 km2 (2.38%).
2. Under the SSP2‐4.5 scenario, HQ, CS, water production, and water purification capacity in Henan Province, however, decreased. Under the SSP5‐8.5 scenario, all three decreased, except for the water production capacity in Henan Province, which increased. The reason may be that a certain amount of arable land, forest land, and grassland is converted into construction land, which triggers the loss of carbon stock and HQ, and the loss area is mainly located around the urban centers of the cities.
3. The spatial distribution pattern of the CES Index shows similarities under different scenarios, with low CES values in construction land and high values in forest and grassland areas. Under the SSP2‐4.5 scenario, the largest decline in CES is observed, primarily due to the encroachment of urban and rural settlements on forested land. Notably, the CES in cities such as Zhumadian, Xinxiang, and Zhoukou experiences significant decreases, highlighting the need for enhanced land use regulation in these areas. In contrast, under the SSP5‐8.5 scenario, the majority of CES increases are observed in Henan Province. However, the average CES in 2050 is still lower than that in 2020, indicating the necessity for government intervention in urban development before 2050 to ensure the sustainable management of ESs.

## Author Contributions

**Jincai Zhang:** conceptualization (equal), writing – original draft (lead), writing – review and editing (lead). **Ling Li:** data curation (equal), formal analysis (equal), resources (equal). **Qingsong Li:** data curation (equal), formal analysis (equal), supervision (equal). **Weiqiang Chen:** investigation (equal), project administration (equal). **Junchang Huang:** validation (equal), visualization (equal). **Yulong Guo:** software (equal), validation (equal). **Guangxing Ji:** formal analysis (lead), funding acquisition (equal), methodology (lead).

## Conflicts of Interest

The authors declare no conflicts of interest.

## Supporting information


Appendix S1.

## Data Availability

The data that support the findings of this study are openly available in the Earth Resources Data Cloud (http://www.gis5g.com/home), OpenStreetMap (https://download.geofabrik.de/), National Geographic Information Resources Catalog Service System (https://www.webmap.cn/), Resource and Environmental Science Data Platform (https://www.resdc.cn/), the World Soil Database (HWSD) (http://webarchive.iiasa.ac.at/Research/LUC/External‐World‐soil‐database/), the Scenario Model Comparison Program of CMIP6 (https://pcmdi.llnl.gov/CMIP6/) and the global 0.25° × 0.25° Land Use Harmonization 2 (LUH2) dataset (https://luh.umd.edu/).
